# Experimental Manipulation of Polyandry in a Marine Gastropod Reveals How the Number of Mates Affects Reproductive Output, Offspring Size, and the Distribution of Paternity Within Broods

**DOI:** 10.1002/ece3.71505

**Published:** 2025-06-01

**Authors:** Alexandra P. Hooks, Scott C. Burgess

**Affiliations:** ^1^ Department of Biological Science Florida State University Tallahassee Florida USA

**Keywords:** fecundity, mate number, *Melongena corona*, multiple mating, paternity analysis, paternity share

## Abstract

Polyandry, where females mate with multiple males, often mediates how ecological and evolutionary forces shape populations, with various explanations for why it occurs. However, these explanations often stem from separate studies on model species, field observations, or lab experiments. Given polyandry's potential context‐dependent effects, it is crucial to design studies that concurrently test multiple hypotheses within wild populations. Therefore, we conducted two experiments over 2 years that experimentally manipulated the number of males a female mates with in the marine gastropod, the Florida crown conch ( *Melongena corona* ). We tested if experimentally increasing polyandry leads to more offspring, larger offspring at hatching, and broods with greater variation in offspring size and higher genetic diversity. We also investigated paternity skew, the effects of mate order, male size, and copulating time on paternity. We genotyped 3157 offspring from 20 mothers to quantify paternity share at hatching. We found that females mating with more males did not produce more offspring or larger offspring than monandrous females at the embryo or hatching stage. However, multiple mating increased within‐brood variation in offspring size at hatching, possibly as a response to exploitative intracapsular competition for oxygen in mixed broods or sire effects. Paternity share within broods at hatching was skewed, rather than evenly distributed, resulting in a lower effective number of sires compared to the number of mates. Paternity share per brood declined with mate order and increased with copulation duration but was unaffected by male size. Overall, the commonly hypothesized consequences of polyandry were not observed in our experiments. Instead, we hypothesize that multiple mating in this species arises from convenience polyandry or mate encounter rates.

## Graphical abstract

We conducted two experiments on a wild population over 2 years, manipulating the number of males copulating with females to assess whether polyandry influences reproductive output, offspring size, or size variability. We also genotyped 3157 offspring from 20 mothers to measure paternity share at hatching.

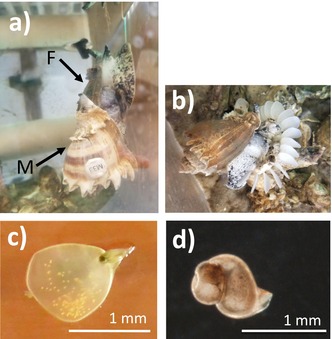

## 1 Introduction

Polyandry, where females mate with two or more males over a single reproductive bout, has attracted much attention because it occurs in many different types of species, varies among individuals and populations within species (Taylor et al. 2014), and has a long list of potential explanations for what causes it (Arnqvist and Nilsson 2000; Jennions and Petrie 2000; Zeh and Zeh 2001; Simmons 2005). Polyandry can also positively or negatively affect population viability by mediating reproductive output and genetic diversity (Simmons 2003; Lotterhos 2011; Holman and Kokko 2013). Despite the consequences polyandry can have on a population, multiple hypotheses for the costs and benefits of polyandry need to be evaluated in wild populations and evaluated with manipulative lab experiments paired with field observations.

Polyandry often increases maternal fitness, suggesting that female fitness is not always limited by egg production (Garant et al. 2005; Neff and Svensson 2013; Yasui and Garcia‐Gonzalez 2016) and that any costs of multiple mating do not always outweigh the benefits (e.g., Fowler and Partridge 1989; Hurst et al. 1995; Crudgington and Siva‐Jothy 2000). In insects, multiple mating can stimulate increased egg production or increase fertilization rates from nuptial gifts, parental care, or to replenish sperm when it is limiting, which may also depend on female condition (Ridley 1988; Arnqvist and Nilsson 2000). In the case of sperm limitation, the benefits of multiple mates may diminish after a certain number of mates are encountered, necessitating manipulations of a range of mate numbers, rather than a single number for multiple mating (Arnqvist et al. 2005). Similarly, multiple mating may benefit larger females more than smaller females since larger females tend to produce more eggs, so are more likely to be sperm limited (MacDiarmid and Butler 1999; Lim et al. 2014). This necessitates the consideration of female size in studies of polyandry. In contrast to these direct material benefits, the benefits of polyandry in other species are thought to arise indirectly.

Indirect, or genetic, benefits of polyandry are driven by several non‐mutually exclusive mechanisms. One proposed mechanism involves mate choice. Females may accept additional matings when their previous mates are deemed to be of inferior genetic quality (Yasui 1998; Jennions and Petrie 2000; García‐González and Simmons 2005; Firman 2011). Similarly, polyandry can allow mate choice to occur after copulation through the differential utilization of sperm from some males over others (Eberhard 1996). Pre‐ and post‐copulatory mate choice through polyandry may also reduce the risk of genetic incompatibilities from unfavorable combinations of parental genotypes that reduce offspring viability, such as occurs from inbreeding (Tregenza and Wedell 1998; Duthie et al. 2016). A second proposed mechanism for the indirect benefits of polyandry involves sperm competition (Simmons 2005). When sperm from multiple males overlap at the site of fertilization, sperm from potential sires compete for fertilizations, and high‐quality males can be better sperm competitors and sire more offspring (Tregenza and Wedell 2000; Hosken et al. 2003; McCullough et al. 2017). Importantly, all these mechanisms should increase biases in paternity after mating and generally lead to offspring with phenotypes that increase survival after fertilization compared to offspring from monandrous females.

Indirect benefits of polyandry can also involve genetic diversity, which in contrast to the previous mechanisms, is not expected to bias paternity but instead lead to even paternity share (Teng and Kang 2007; McLeod and Marshall 2009; Jaffé et al. 2012). Broods of offspring produced by mothers with multiple sires have higher genetic diversity, resulting in offspring within broods being less related to each other (more paternal‐half sibling groups) compared to those from mothers that have mated with a single male (all full siblings). Genetic diversity can increase offspring survival through complementarity effects, where genetically different individuals compete less through niche partitioning in terms of resource utilization (Kamel et al. 2010). For example, in cases where offspring develop together inside egg capsules, variation in developmental rates among larvae from different sires could offset the overlap of times with peak oxygen demands (Moran and Emlet 2001). Indiscriminate mating could also increase fitness by increasing genetic diversity in broods to reduce the risks of non‐viable offspring (i.e., bet‐hedging) (García‐González et al. 2015; Yasui and Garcia‐Gonzalez 2016). For maximized benefits of genetic diversity for mothers, sires should be equally represented when an unpredictable offspring environment makes the “best” sire unknown at mating.

Regardless of the mechanism, many prior hypotheses on the benefits of polyandry predict that polyandry should increase the number of viable offspring, either through fecundity or offspring survival. Furthermore, the extent to which polyandry leads to biases in paternity can be used to understand which types of mechanisms might be more likely. Gastropods that encapsulate embryos are a particularly useful group to study polyandry because offspring from multiple sires commonly develop within the same egg capsule, providing the arena for any costs and benefits of polyandry to play out. For instance, encapsulated offspring can compete for oxygen, space, and nutrients, but these challenges might be mitigated through resource partitioning, cannibalism, or other intra‐brood interactions that can vary with sibling relatedness (Kamel and Williams 2017). In that context, Florida crown conch ( *Melongena corona* ) is a particularly relevant and tractable species to study polyandry as the number of mates can be experimentally manipulated in the lab and paternity identified using genetic markers. Previous field studies have identified the potential for a large number and range of sires (2–19) within a single capsule (Hooks and Burgess 2024). Here, we experimentally tested the hypothesis that increasing the number of males a female copulated with will produce: (1) more offspring, (2) larger offspring at hatching, (3) broods with greater variation in size at hatching, and (4) broods with a greater number of sires represented at hatching. For Hypothesis 1, we first considered mate numbers ranging from 1 to 5 to test if the number of mates had an additive or non‐additive effect on female reproductive output (2018). Then in a second, larger experiment (2019), we compared females mated with one or four males. We considered multiple aspects of reproductive output, such as how the total output was distributed within and among egg capsules and egg strings. For Hypothesis 4, we were interested in the effective number of sires, which indicates the extent to which a single sire dominates the brood or if paternity is evenly distributed among sires. For Hypothesis 4, we also tested how mate order, male size, and copulation time affected the proportion of offspring a male sired in mixed broods. We also provide the first detailed observations of mating behavior in the lab over the 2 years of this study.

## 2 Methods

### 2.1 Study System

**FIGURE 1:**
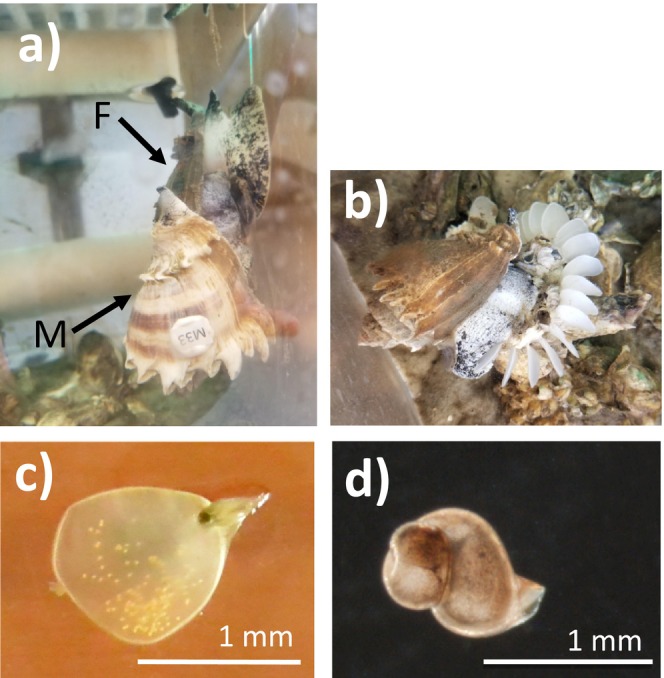
(a) A male (M) and female (F) copulating in lab. (b) A female laying an egg string with multiple egg capsules (Hooks and Burgess 2021). (c) A single egg capsule packaged with offspring (small yellow dots inside capsule) early (~5 days) in development. (d) A newly emerged hatchling.

The Florida crown conch ( *Melongena corona* ; Gastropoda, Melongenidae) occurs in intertidal and shallow subtidal habitats along the coasts of Florida and eastern Alabama (Hathaway 1958; Menzel and Nichy 1958; Hathaway and Woodburn 1961; Woodbury 1986; Bowling 1994). Individuals are gonochoristic and mating occurs via copulation (Figure 1a). Females lay strings of coin‐shaped egg capsules (averaging 10 egg capsules per string but can range from ~5 to 25 in the field, Figure 1b) with each egg capsule containing ~40–200 offspring (Figure 1c). Embryos develop inside egg capsules for ~16–35 days until they emerge as crawl‐away larvae (Figure 1d) and metamorphose within a few days (Hooks and Burgess 2021). Egg capsules of the crown conch do not contain nurse eggs; there is no intracapsular cannibalism (Hooks, unpublished data), and embryos do not use intracapsular fluid for nutrition (Noriega and Miloslavich 2010). At our study site, mating and egg laying occur during the warmer months of March–August. The study population was located in the oyster and seagrass habitat adjacent (west) to the Florida State University Coastal and Marine Laboratory (29°54′57.87′′ N 84°30′38.66′′ W). Mating manipulations were carried out in 2018 and 2019 using different individuals from the same population.

### 2.2 Experimental Design

We avoided using sexually immature individuals by collecting crown conch above certain size thresholds. Females were operationally defined by the absence of a penis and the presence of a gonopore. Males were operationally defined by the presence of a penis and the absence of a gonopore. Females were collected if they measured ≥ 55 mm in length; males were collected if they measured ≥ 35 mm. Different size thresholds were necessary because females are generally larger than males in this species (Hooks, personal observation). In both 2018 and 2019, all males and females were collected from the same location and tagged with unique identifiers using marine epoxy and waterproof paper labels. No individuals were reused across years. Males were retired after their first mating event, and both sexes were fed live oysters ad libitum throughout the duration of each experiment.

#### 2.2.1 Experiment 1 (2018)

Twenty‐eight adult females were collected from the field in March 2017 and kept in individual 12.5‐L tanks with flow‐through seawater and fed oysters ad libitum until May 2018 (14 months). Most females stopped depositing eggs after 4 months, and the few that continued produced only one or two additional egg strings composed entirely of unfertilized egg capsules, suggesting that sperm stores were depleted prior to the mating experiment. Males were collected from the field in February 2018, before the start of the mating season, and maintained on a flow‐through wet table until used in the experiment. Females were randomly allocated to one of five mating treatments: one male (*n* = 11 females), two males (*n* = 3 females), three males (*n* = 2 females), four males (*n* = 3 females), or five males (*n* = 3 females). Mating of higher numbers of males was attempted, but females sometimes started laying their first egg string after a single mate and four males being the highest average of mates before females started egg laying. Mating attempts with higher numbers of males were made, but females sometimes began laying their first egg string after a single mating. The highest average number of mates a female would accept before laying eggs was four. After their first mating, males were retired from the mating trails and were not used again. In addition, five females were kept in isolation and never mated to test if females could be using stored sperm acquired 14 months prior before they were collected from the field. Isolated females produced egg capsules for only 4 months in isolation, after which they no longer produced fertilized egg strings. Only one female died during the experiment before laying offspring and was thus removed from all analyses.

For each mating, a single male was placed into a female's tank at any one time. In most cases, males approached females within the first 5 min of being placed in a female's tank and attempted to copulate. Females would either allow the copulation to occur (Figure 1a) or would refuse the male by clamping down their shell to prevent copulation. If a male did not mate within 4 h, he was removed and replaced with another randomly chosen male. Males that did not successfully copulate were presented to another female in another week of the experiment. All males eventually copulated with a female and then were retired from the experiment. Copulation was confirmed visually by observing the penis inserted under the female's shell (we used genetic markers to check if copulation led to viable hatchlings for a given sire). Males were removed after visible copulation ended and the pair separated. Another male was then added according to the treatment of each female. Mating observations were made every 30 min to ensure that females did not re‐mate with the same male. Females mated up to twice per day (i.e., up to two males) and completed mating at the prescribed number of mates within 5 days. All females mated within a 2‐week period in May 2018. Females laid egg strings within 1–12 days after mating and only the number of hatchlings and egg capsules were counted for the first egg string laid.

Once laid, egg strings were removed from the female's tank, labeled based on mother identity, and placed on a wet table with flow‐through seawater. When offspring were close to hatching, as observed by the presence of a fully formed foot in the encapsulated offspring, egg capsules were cut from the egg string and placed individually into 50 mL conical tubes filled with 29 ppt filtered seawater (seawater was collected from the field and filtered with an activated carbon and coarse foam water filtration system). Tubes were then placed in an incubator chamber at 28°C, which is within the optimal temperature range (27°C–30°C) for egg capsule development (Hooks, personal observation). Isolating individual egg capsules before hatching prevented protist outbreaks. The seawater in each tube was replaced 6 days per week. Egg capsules were checked for hatchlings every day. Once the first hatchling emerged from an egg capsule (Figure 1d), the total number of hatchlings was counted after cutting open the egg capsule. It was not feasible to count hatchlings in every egg capsule because females laid too many egg capsules at once. As a result, half of the egg capsules were kept and counted from each mother. Egg capsules were randomly chosen within each egg string, and each mother had a minimum of 10 egg capsules per egg string.

#### 2.2.2 Experiment 2 (2019)

Females for the second experiment were collected in February 2019, before the start of the mating season, and kept in isolation until the start of experiments in late April 2019 (~3 months). Males were collected in groups between March and May 2019 and kept together before being used in mating trials. Sixty females were collected and maintained in isolated mesh cages placed in a flow‐through wet table for the duration of the experiment (February–August).

Following the same procedure as in 2018, individual mating pairs were placed together in 12.5‐L tanks to allow for ease of observations every 30 min. Once copulation was complete, males were replaced with a new male. If no copulation occurred within 4 h, males were replaced with a new male. Twenty‐six females mated with one male while 27 females mated with four males, except for three females that accidentally mated with five or more males. Due to the high number of females, not all females could be mated at the same time. As a result, matings were staggered over an 8‐week period starting at the end of April 2019. Six to fourteen females were mated within a given week. In each week, roughly equal numbers of females were mated with one or four males. In Hooks and Burgess (2024), we found no seasonal or interannual differences in the total reproductive output from egg strings laid early versus late in the reproductive season, supporting the validity of staggering mating events in the laboratory without compromising reproductive potential.

Once a female laid an egg string, egg capsules were individually placed in 100 mL plastic containers with 500 μm mesh openings that prevented hatchlings from escaping the container but allowed water flow. Egg capsules in mesh containers were maintained in two 379‐L tanks with UV‐radiated artificial seawater that flowed at 340 L per hour. The water was maintained at 28°C and 29 ppt. UV‐radiated seawater was used to decrease protist load and increase egg capsule survival.

To determine if any mortality during intracapsular development depended on the number of mates, offspring were counted at the embryo and hatching stage. Counting embryo number is a destructive process that requires the egg capsule to be cut open. Therefore, 70% of egg capsules on a mother's egg string were randomly allocated to be counted at either the embryo or hatchling stage (35% of egg capsules per mother at each life stage). The number of embryos was counted 4 days after being laid, at which time offspring were multicellular but had not developed into a larva. The number of hatchlings was counted the day that the egg capsules opened, allowing hatchlings to emerge.

Shell length was recorded for 35% of hatchlings, or up to 40 individuals, per egg capsule. To measure shell length, hatchlings were photographed with a camera‐mounted dissecting microscope at a total magnification of 200×, then the longest axis of the shell was measured using Image Insight Pro (v. 9.1). In total, the sizes of 5393 hatchlings were measured (44–278 hatchlings measured per mother).

To quantify the number of egg strings a female laid, 56 mothers that had finished mating were isolated until the end of the reproductive season, and the number of egg strings and egg capsules per mother was recorded. The number of offspring per capsule was only counted in the first egg string.

### 2.3 DNA Paternity Analysis

Parents and offspring from the multiple mating treatment in the 2019 experiment were genotyped at six microsatellite loci to estimate the distribution of paternity per mother. From each mother that laid, 2–5 egg capsules were sampled (representing an average of 67% of egg capsules per mother). From each sampled capsule, we sampled 25%–35% of randomly selected offspring, and a minimum of 16 offspring per capsule. Overall, we genotyped 40–172 offspring per mother from 20 mothers, resulting in a total of 3157 offspring genotyped in the polyandry treatment. Prior work using naturally laid egg strings collected from the field demonstrated that smaller sample sizes (~8% of offspring per mother) are sufficient to detect high levels of polyandry (Hooks and Burgess 2024). Mothers that mated once did not have their offspring genotyped since there was no evidence in 2018 that females used stored sperm acquired prior to the experiment.

DNA was extracted by placing the entire hatchling in a solution of 150 μL of Chelex 100 Resin (Bio‐Rad) and 3 μL of 25 mg/mL concentrated proteinase K (IBI Scientific). Thermocycler conditions for extracting DNA: 55°C for 60 min followed by 95°C for 15 min. DNA from parents was extracted under the same conditions but from a small piece of tissue from their foot.

All extractions were amplified for six microsatellite loci in a single multiplex reaction (microsatellite information provided in Hooks and Burgess 2024). Polymerase chain reaction (PCR) was performed in a 10 μL volume reaction with 1 μL of undiluted DNA, 1.2 μL of primer stock mix, 2 μL of 0.05 mg/mL BSA (New England Biolabs), 0.8 μL of sterile deionized water, and 5 μL of Qiagen Multiplex PCR master mix (Qiagen). All forward primers were tailed with universal primers Tail A‐D described in (Blacket et al. 2012) and tagged with fluorescent dyes of either VIC, NED, FAM, or PET. The primer stock mix included a 1:1:2:2:1:1 ratio of Mcor3010, Mcor4853, Mcor6632, McoA4, Mco2, and Mco10 primers, respectively, each at a 20 μM concentration. The primer stock mix included the forward primer with the respective universal at equal quantities and the reverse primer at equal quantities of the forward and universal combined (i.e., 1 μL forward primer, 1 μL universal tagged primer, and 2 μL reverse primer each at 20 μM). Cycling conditions for PCRs were: 95°C for 15 min followed by 32 cycles of 94°C for 30 s, 57°C for 90 s, and 72°C for 90 s, followed by a final extension time of 72°C for 10 min.

Individuals were genotyped using fragment analysis. Per individual sample, fragment analysis used 1 μL of PCR product, 0.02 μL of Liz‐500 size standard, and 10 μL of Hi‐Di formamide (Applied Biosciences). Fragment analysis was done using a 3730 Genetic Analyzer at the Biology Core Facility at Florida State University. Fragment lengths were scored manually using Geneious v9.1.8 (Biomatters Ltd).

Offspring, with known mothers and a set of candidate sires based on the experimental matings, were assigned paternity using Colony v2.0.6.4 (Jones and Wang 2010). Input parameters for Colony were polygamous for both sexes, dioecious, with no inbreeding present, diploid, one long run of the full‐likelihood model, high likelihood precision, and no updating of allele frequencies. Error rates were set at 0.01, a strong prior chosen due to the mating design nature of the data, estimated paternal sibship size was set to 40, and maternal sibship size to 150. The mismatch threshold for mothers was set at zero.

Colony successfully assigned 98% of offspring to a father with 100% probability. The remaining 2% of offspring were assigned paternity with a probability ranging from 53% to 99%. For these cases, we manually and unambiguously verified the assignments, which was possible due to the high polymorphism of the microsatellite markers used (Hooks and Burgess 2024) and the controlled mating design in which the genotypes of the four candidate fathers and the mother‐offspring relationship were known. No novel alleles were detected during paternity assignment, further supporting the accuracy of the assignments to the known candidate fathers.

### 2.4 Statistical Analyses

Analyses were performed in R version 3.6.3 (R Core Team 2018) using generalized models (glmm) implemented using the “glmmTMB” package. Each year was analyzed separately. For both years, we included the number of mates (1–5 in 2018; 1 or 4–6 in 2019) as a categorical factor. For the 2018 dataset, treating the number of mates as a categorical variable allowed for any non‐linear patterns across the range of mates to be detected. Mother size (shell length) was included as a continuous covariate to account for the possibility that larger mothers produce more offspring or larger offspring. Interactive effects for mother size were assessed in each model and when not significant, were excluded. Residual diagnostics were assessed using DHARMa (Hartig 2022) and significance was assessed at *α* = 0.05.

#### 2.4.1 Offspring Numbers and Egg Capsule Counts (2018 Hatchlings and 2019 Embryos and Hatchlings)

The effect of mate number on the mean number of embryos and hatchlings per egg capsule was tested using a Gaussian model with treatment and mother length as fixed factors and offspring number log‐transformed to meet Gaussian assumptions. Estimated mortality from the embryonic stage to hatching was assessed by comparing the average number of offspring per egg capsule at the embryo stage compared to the hatchling stage using a mixed‐effects model with a Gaussian distribution (numbers were log transformed to meet model assumptions). Offspring type (embryo or hatchling) and treatment (one or four mates) were included as fixed effects, and mother identity was included as a random effect. A statistically significant effect of offspring type on offspring number per capsule would indicate mortality during embryonic development. The influence of mate number on the number of egg strings per mother (2019) and egg capsules per egg string (2018) was tested using Poisson distributions. The total number of egg capsules across all egg strings in 2019 was modeled with a negative binomial distribution. Hypothesis testing used chi‐squared log‐likelihood ratio tests.

#### 2.4.2 Probability of Laying Egg Strings and Total Reproductive Output (2018 and 2019)

All females laid egg strings in 2018, whereas only 62% of females (33 out of 53) laid egg strings in 2019. The difference is likely due to females being fed continuous food for a longer period of time in 2018 compared to 2019. For females in 2019, the effect of the number of mates on the probability of laying eggs was tested using a binomial generalized linear model.

Total reproductive output of each mother was estimated as the mean number of offspring per egg capsule (estimated from 2 to 5 capsules per mother on the first egg string) multiplied by the total number of egg capsules that were counted across all egg strings laid during the experiment. This calculation assumes that the mean number of offspring per egg capsule is similar across egg strings from the same mother. For 2018, reproductive output included all egg capsules laid only on the first egg string, while in 2019, reproductive output included all egg strings laid during the entire reproductive season.

For the 2018 data, the effects of the number of mates on reproductive output at the hatching stage was tested using a Gaussian model with reproductive output being log transformed for a better model fit. In 2019, reproductive output was estimated separately for embryo and hatching stages. Because not all females laid egg capsules in 2019, we used a hurdle model to include the structural zero counts. A hurdle model is a two‐part model, where the first part models the probability that a positive count is observed using a binomial model with a logit link function, and the second part models abundance using only the non‐zero data and a zero‐truncated negative binomial distribution (“truncated_nbinom1”) and a log link function (“zi=~treatment*mother.length”). Therefore, reproductive output is a product of the conditional mean (i.e., mean of the non‐zero offspring counts) and the probability of laying eggs.

#### 2.4.3 Size and Coefficient of Variation in Size at Hatching (2019)

To examine the effect of mate number on offspring size and its coefficient of variation (CV) at hatching, we used linear mixed‐effects models. Fixed effects included the number of mates, maternal length, and developmental time (days spent inside the egg capsule). Random effects were mother identity and egg capsule nested within mother identity when testing offspring size, and only mother identity for offspring size variation. Development time was included to account for its potential effect on offspring size. We partitioned variation in offspring size by calculating the variance components for each random effect relative to total variance.

Offspring size CV within each egg capsule was calculated as the standard deviation divided by the mean size, then averaged per mother for analysis. Multiple mixed models were tested for additive and interactive effects of all fixed factors, with model selection based on AICc using the aictab function in R. The coefficients of the best model were evaluated for significance using a type III ANOVA for both offspring size and size variation.

#### 2.4.4 Distribution of Paternity (2019)

We compared the number of experimentally determined mates with the number of sires at hatching, estimated from paternity analysis, for females mated to four males in the 2019 experiment. To quantify the degree of paternity share within broods, we calculated the effective number of sires using the Shannon diversity index, as implemented in the “vegan” package (v. 2.5‐6) in R, and then exponentiated (Jost 2006). This exponentiated value reflects the number of sires and their relative representation at hatching: if all mates contribute equally, the effective number of sires is 4; as paternity share becomes more skewed, it approaches 1.

To test whether the number and effective number of sires differed from the number of mates, we conducted one‐tailed *t*‐tests, with the null hypothesis that the number of mates was 4. We used a Gaussian model to assess the effect of the effective number of sires on total reproductive output for polyandrous mothers. To evaluate the impact of maternal length on the number and effective number of sires at hatching, we applied negative binomial generalized linear models.

To investigate the effects of mate order, male size, and copulation time on the proportion of offspring sired by each male, we fitted separate mixed‐effects models with a beta distribution and logit link function using the glmmTMB package. The response variable in all models was the proportion of offspring sired by a particular sire, with mother identity modeled as a random effect. Hypothesis tests were performed using chi‐squared log‐likelihood ratio tests. Since no interactive effects were found between treatment and mother length, only additive effects are reported.

## 3 Results

### 3.1 Observations of Mating Behaviors in the Lab

Both sexes initiated mating behavior by approaching the other, although mating was most often initiated by the male. In many trials, interactions between the sexes began within 30 min of being placed in the tank. When males and females were placed together, one of several distinct interaction patterns was observed: (1) both individuals remained stationary (rare), (2) the male approached the female and initiated copulation, (3) the male attempted copulation, but the female either moved away or clamped its shell to the tank, which prevented copulation, (4) after the female moved away or clamped down, the male attached his foot to the female's shell (usually at the apex), twisted his foot around until the female was dislodged, then attempted copulation, (5) the female approached the male, but the male did not respond or emerge from his shell during the trial (rarely observed), (6) the female approached the male and initiated copulation (rare).

In the vast majority of cases, males initiated copulation, and females responded either by copulating with males or moving in a way that prevented copulation. Copulation was visually observed when the male's penis was inserted beneath the female's shell. Mating events averaged approximately 3 h but could last up to 10 h. During copulation, mating pairs remained immobile. Copulation was often terminated by females moving away, with males sometimes attempting to remate a few minutes after detachment. After copulation ended, copulation with a newly introduced male was observed within 30 min; however, it often took an hour or more before copulation started. Sometimes, females in the experiment did not engage in copulation 1 day, but then would engage the following day. Some females would move away from males or clamp down on their shells which prevented copulation for several consecutive days. Egg laying typically began within the first week after the mast mating event and ranged from 2 days after the last mating event to up to 2 weeks. It took approximately 1–2 h for females to finish laying an egg string once started (see also Hulse et al. 2023).

### 3.2 Offspring Numbers and Egg Capsule Counts (2018 Hatchlings and 2019 Embryos and Hatchlings)

#### 3.2.1 2018 Results

**FIGURE 2:**
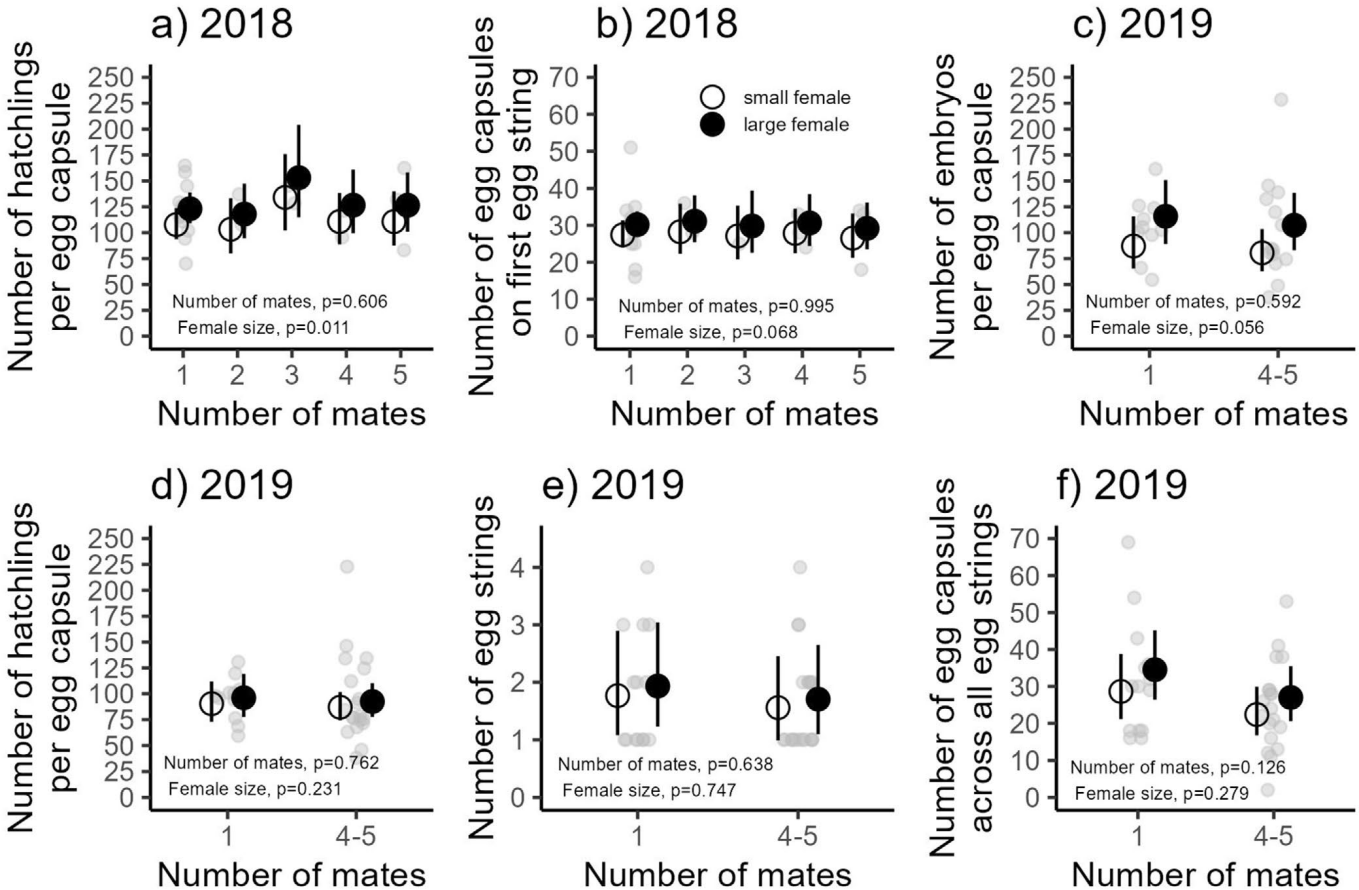
The effects on number of mates on (a) average number of hatchlings per egg capsule in 2018, (b) average number of egg capsules on the first egg string in 2018, (c) average number of embryos per egg capsule in 2019, (d) average number of hatchlings per egg capsule in 2019, (e) average number of egg strings laid across the entire reproductive season in 2019, and (f) average number of egg capsules across all egg strings laid in 2019. While mother size was modeled as continuous variable, the figures here show a representative large female (solid circles; average shell length of 71.55 mm representing the top 20% of largest females collected in 2019) and small female (open circles; average shell length of 60.80 mm representing the bottom 20% of smallest females collected in 2019). Black lines show 95% CIs, and light gray symbols show individual females.

Hatchling numbers per egg capsule did not differ between females that mated with one versus multiple males (*χ* ^2^ = 2.71, df = 4, *p* = 0.606; Figure 2a; Table 1). Mothers produced an average of 119.7 hatchlings per capsule (95% CI: 109.67–130.65), with hatchling number increasing by 2% (95% CI: 1%–4%) for every 1 mm increase in female size.

**TABLE 1:** Parameter estimates of Gaussian models testing sire number and mother length on offspring number response variables for 2018.

Parameter	Hatchling number per egg capsule	Egg capsules on first egg string	Reproductive output (# hatchlings)
Intercept (*⍺*)	3.21 (2.00–4.42, 95% CI)	2.26 (1.04–3.47, 95% CI)	5.43 (2.77–8.09, 95% CI)
2 Mates (1)	−0.04 (−0.28–0.19, 95% CI)	0.03 (−0.20–0.26, 95% CI)	0.03 (−0.49–0.54, 95% CI)
3 Mates (1)	0.22 (−0.06–0.49, 95% CI)	−0.01 (−0.30–0.28, 95% CI)	0.24 (−0.36–0.85, 95% CI)
4 Mates (1)	0.03 (−0.21–0.26, 95% CI)	0.01 (−0.23–0.26, 95% CI)	0.08 (−0.44–0.59, 95% CI)
5 Mates (1)	0.03 (−0.20–0.26, 95% CI)	−0.03 (−0.27–0.21, 95% CI)	0.00 (−0.51–0.50, 95% CI%)
Mother length (2)	**0.02 (0.00–0.04, 95% CI)**	0.02 (0.00–0.03, 95% CI)	**0.04 (0.00–0.07, 95% CI)**

*Note:* Bolded Mother length for hatchling number and reproductive output.

Similarly, the number of egg capsules in the first egg string did not vary with mate number (*χ* ^2^ = 0.21, df = 4, *p* = 0.995; Figure 2b; Table 1). Mothers laid an average of 29 capsules (95% CI: 27.05–31.58), and this count was not significantly associated with female size (*χ* ^2^ = 3.33, df = 1, *p* = 0.068).

#### 3.2.2 2019 Results

Mothers produced an average of 96.68 embryos per egg capsule (95% CI: 83.01–112.59), with no significant effect of mate number on embryo count (*χ* ^2^ = 0.29, df = 1, *p* = 0.592; Figure 2c; Table 2). Embryo number increased by 3% (95% CI: 1%–5%) for every 1 mm increase in female size.

**TABLE 2:** Parameter estimates for treatment and mother length on offspring number response variables for 2019.

Response variables	Model	Parameters		
		Intercept (*⍺*)	Treatment (1)	Mother length (2)
Number of embryos in an egg capsule	Gaussian (log normal)	2.85 (1.01–4.68, 95% CI)	−0.08 (−0.36–0.21, 95% CI)	0.03 (0.00–0.05, 95% CI)
Number of hatchlings in an egg capsule	Gaussian (log normal)	4.15 (3.50–4.81, 95% CI)	−0.04 (−0.29–0.21, 95% CI)	0.01 (0.00–0.02, 95% CI)
Total number of egg strings	Negative binomial	0.02 (−3.63–3.67, 95% CI)	−0.13 (−0.65–0.40, 95% CI)	0.01 (−0.05–0.06, 95% CI)
Total number of egg capsules across all egg strings	Poisson	0.02 (−3.63–3.67, 95% CI)	−0.13 (−0.65–0.40, 95% CI)	0.01 (−0.05–0.06, 95% CI)
Probability to lay	Binomial	0.86 (−6.72–8.45, 95% CI)	0.65 (−0.55–1.84, 95% CI)	−0.01 (−0.13–0.10, 95% CI)
Reproductive output (embryos)	Hurdle	5.36 (3.02–7.70, 95% CI)	−0.30 (−0.64–0.05, 95% CI)	**0.04 (0.01–0.08, 95% CI)**
Reproductive output (hatchlings)	Hurdle	4.92 (2.36–7.47, 95% CI)	−0.22 (−0.58–0.13, 95% CI)	**0.05 (0.01–0.08, 95% CI)**

*Note:* Parameter estimates are in the link scale of the model (i.e., binomial models are on the logit scale) and not transformed. Bolded Mother length for reproductive output for both embryos and hatchlings.

Hatchling numbers also did not differ between monandrous and polyandrous females (*χ* ^2^ = 0.09, df = 1, *p* = 0.762; Figure 2d; Table 2). Mothers averaged 89.78 hatchlings per capsule (95% CI: 79.52–101.35), with a 1% increase (95% CI: 1%–2%) for every 1 mm increase in female size. There was no significant difference between the number of embryos and hatchlings within a capsule (*χ* ^2^ = 2.56, df = 1, *p* = 0.109) or across treatments (*χ* ^2^ = 0.007, df = 1, *p* = 0.935), indicating overall low embryonic mortality.

The number of egg strings laid did not differ between monandrous and polyandrous females (*χ* ^2^ = 0.22, df = 1, *p* = 0.638; Figure 2e; Table 2). Mothers laid an average of 1.73 egg strings (95% CI: 1.33–2.24), and female size had no effect on egg string count (*χ* ^2^ = 0.10, df = 1, *p* = 0.747).

Total egg capsules laid during the reproductive season were also similar between mating treatments (*χ* ^2^ = 2.34, df = 1, *p* = 0.126; Figure 2f; Table 2). Mothers averaged 27.7 capsules (95% CI: 23.33–32.89), with no significant effect of female size on total capsule number (*χ* ^2^ = 1.17, df = 1, *p* = 0.279).

### 3.3 Probability of Laying Egg Strings and Total Reproductive Output (2018 and 2019)

**FIGURE 3:**
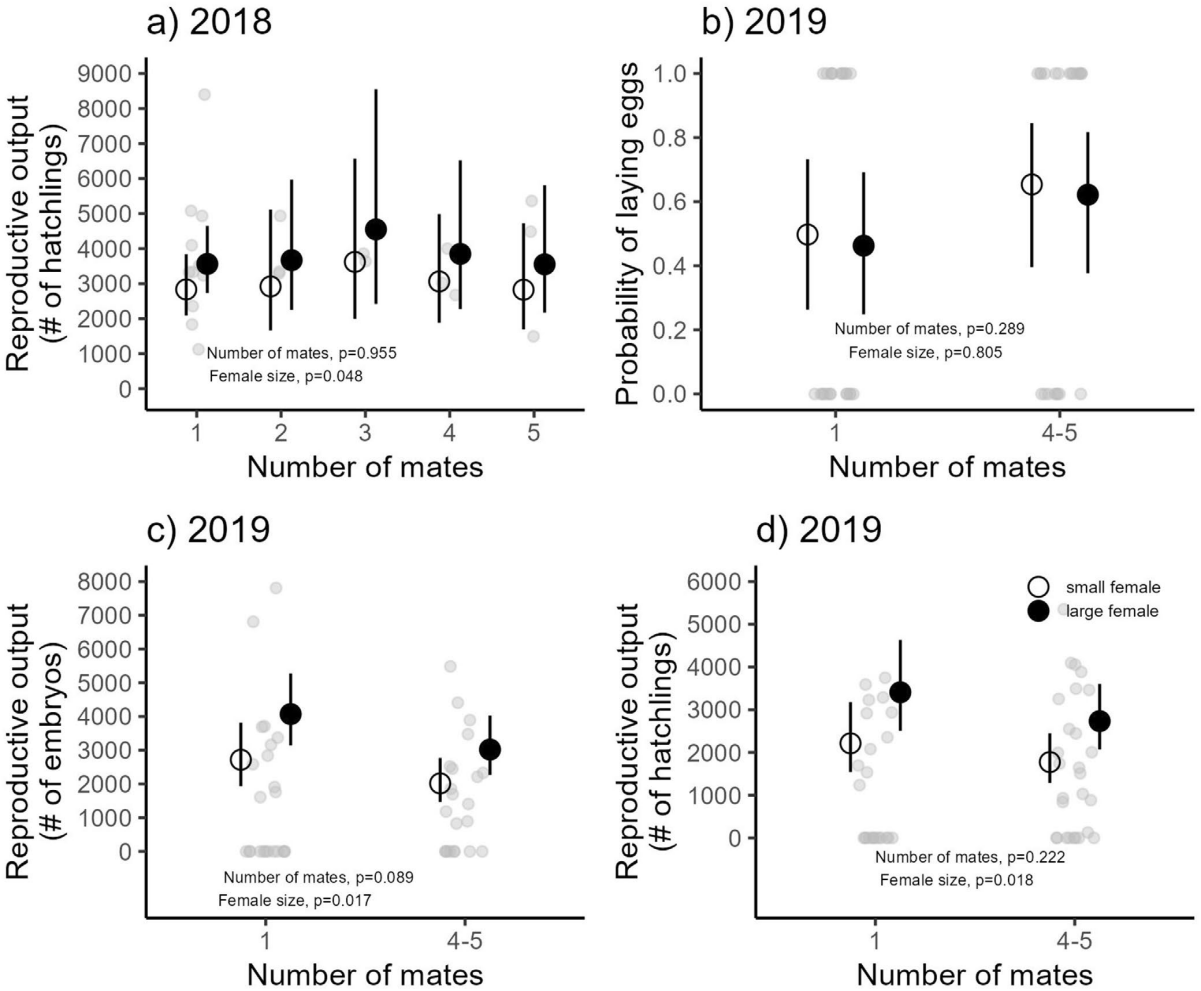
The effect of the number of mates on (a) the estimated number of hatchlings from one egg string in 2018, (b) the probability of females laying eggs in 2019 (note that all females laid in 2018), (c) the estimated number of embryos across the entire reproductive season in 2019, and (d) the estimated number of hatchlings across the entire reproductive season in 2019. While mother size was modeled as continuous variable, the figures here show a representative large female (solid circles; average shell length of 71.55 mm representing the top 20% of largest females collected in 2019) and small female (open circles; average shell length of 60.80 mm representing the bottom 20% of smallest females collected in 2019). Black lines show 95% CIs, and light gray symbols show individual females.

In 2018, mothers averaged 3392 hatchlings (95% CI: 2831–4064) in their first reproductive bout. Hatchling number did not vary with mate number (*χ* ^2^ = 0.67, df = 4, *p* = 0.955; Figure 3a; Table 1), but larger females produced 4% more hatchlings (95% CI: 1%–7%) for each additional 1 mm of size (*χ* ^2^ = 3.91, df = 1, *p* = 0.048).

In 2019, the probability of females laying eggs did not differ between mating with one or multiple males (*χ* ^2^ = 1.125, df = 1, *p* = 0.289; Figure 3b; Table 3). Female size also had no effect on egg‐laying probability (*χ* ^2^ = −0.06, df = 1, *p* = 0.805; Table 3). Mothers produced an average of 2533 embryos (95% CI: 2034–3155) over the reproductive season, with no effect of mate number on embryo production (*χ* ^2^ = 2.899, df = 1, *p* = 0.089; Figure 3c). However, female size significantly influenced embryo output, with a 4% increase (95% CI: 1%–8%) for each additional 1 mm of size (*χ* ^2^ = 5.71, df = 1, *p* = 0.017). The number of mates did not affect hatchling production in 2019 (*χ* ^2^ = 1.49, df = 1, *p* = 0.222; Figure 3d; Table 3). On average, mothers produced 2057 hatchlings (95% CI: 1592–2659), and larger females produced 5% more hatchlings (95% CI: 1%–8%) per additional 1 mm of size (*χ* ^2^ = 5.59, df = 1, *p* = 0.018).

**TABLE 3:** Parameter estimates for linear effects models including treatment, mother length, and development time on offspring size response variables for 2019.

Parameter	Hatchling size	CV of size
Intercept (*⍺*)	816.12 (799.74–832.50, 95% CI)	−0.0212 (−0.002–0.044, 95% CI)
Treatment (1)	2.10 (−18.37–22.57, 95% CI)	**0.0037 (0.0005–0.007**, **95% CI)**
Mother length (2)	2.09 (−7.74–11.93, 95% CI)	−0.0001 (−0.0004–0.0003, 95% CI)
Development time (3)	**−11.40 (−15.17 to −7.62**, **95% CI)**	**0.0003 (0.0001–0.0006**, **95% CI)**

*Note:* Bold parameter estimates indicate significant effects detected.

### 3.4 Size and Coefficient of Variation in Size at Hatching (2019)

**FIGURE 4:**
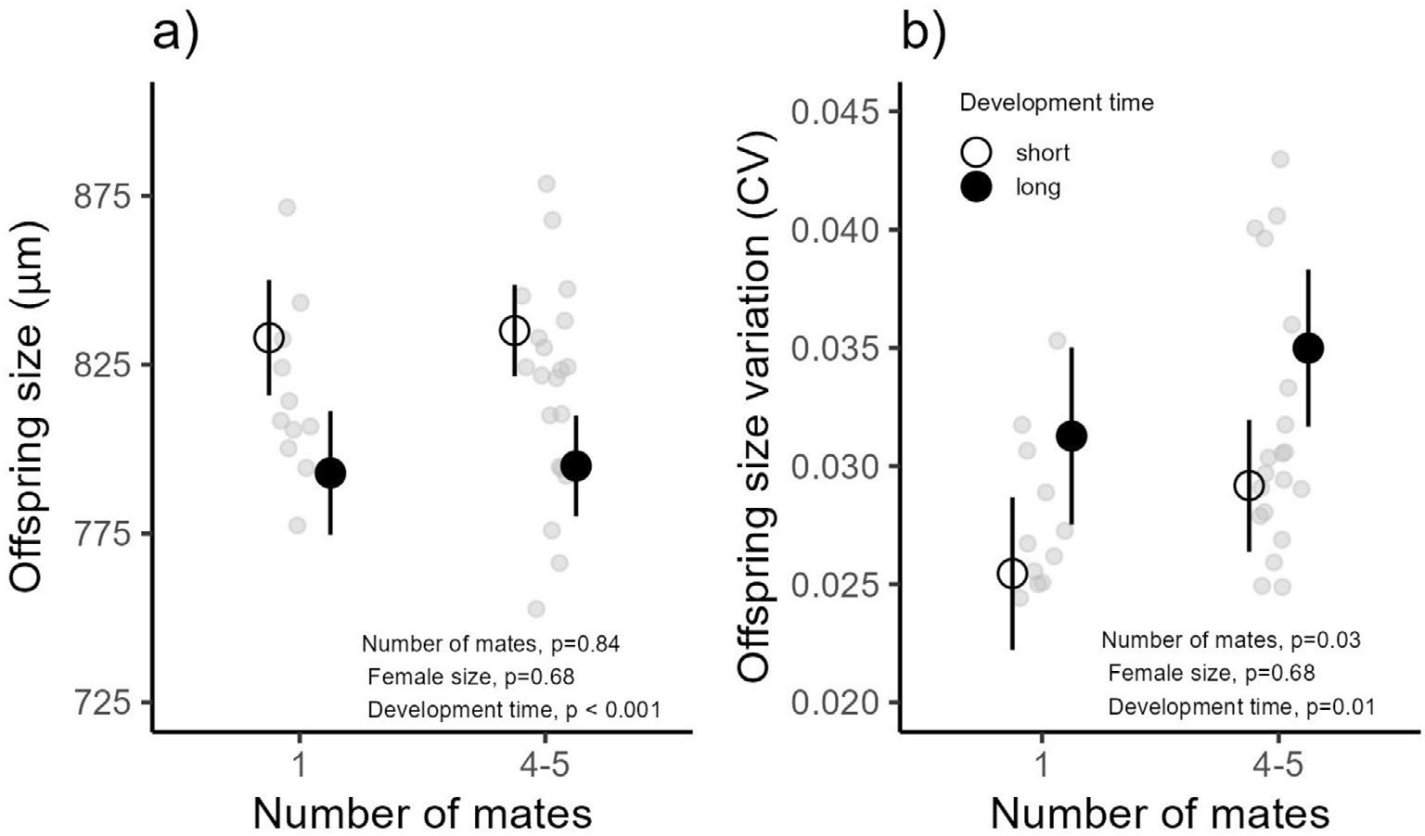
Effects of mate number on offspring traits: (a) offspring size (μm, shell length at hatching) and (b) the coefficient of variation in offspring size. Solid circles represent the average development time of offspring in the longest 20% quartile (those taking the most time to develop), while unfilled circles represent the average development time of offspring in the shortest 20% quartile (those taking the least time to develop). Black lines indicate 95% confidence intervals, and light gray symbols represent individual mothers.

Offspring size decreased with development time (*χ* ^2^ = 35.05, df = 1, *p* < 0.001), but was unaffected by the number of mates (*χ* ^2^ = 0.04, df = 1, *p* = 0.84) or maternal size (*χ* ^2^ = 0.17, df = 1, *p* = 0.68; Figure 4a). The average offspring size was 818.57 μm (95% CI: 808.48–828.66). After accounting for treatment, maternal size, and development time, 42% of the variation in offspring size was attributed to differences among mothers, 19% to variation among egg capsules within mothers, and 38% to variation among hatchlings within each egg capsule. The coefficient of variation (CV) in offspring size per egg capsule increased with the number of mates (*χ* ^2^ = 4.98, df = 1, *p* = 0.03) and with longer development times (*χ* ^2^ = 6.30, df = 1, *p* = 0.01), but was unaffected by maternal size (*χ* ^2^ = 0.17, df = 1, *p* = 0.68) (Figure 4b; Table 3).

### 3.5 Distribution of Paternity (2019)

**FIGURE 5:**
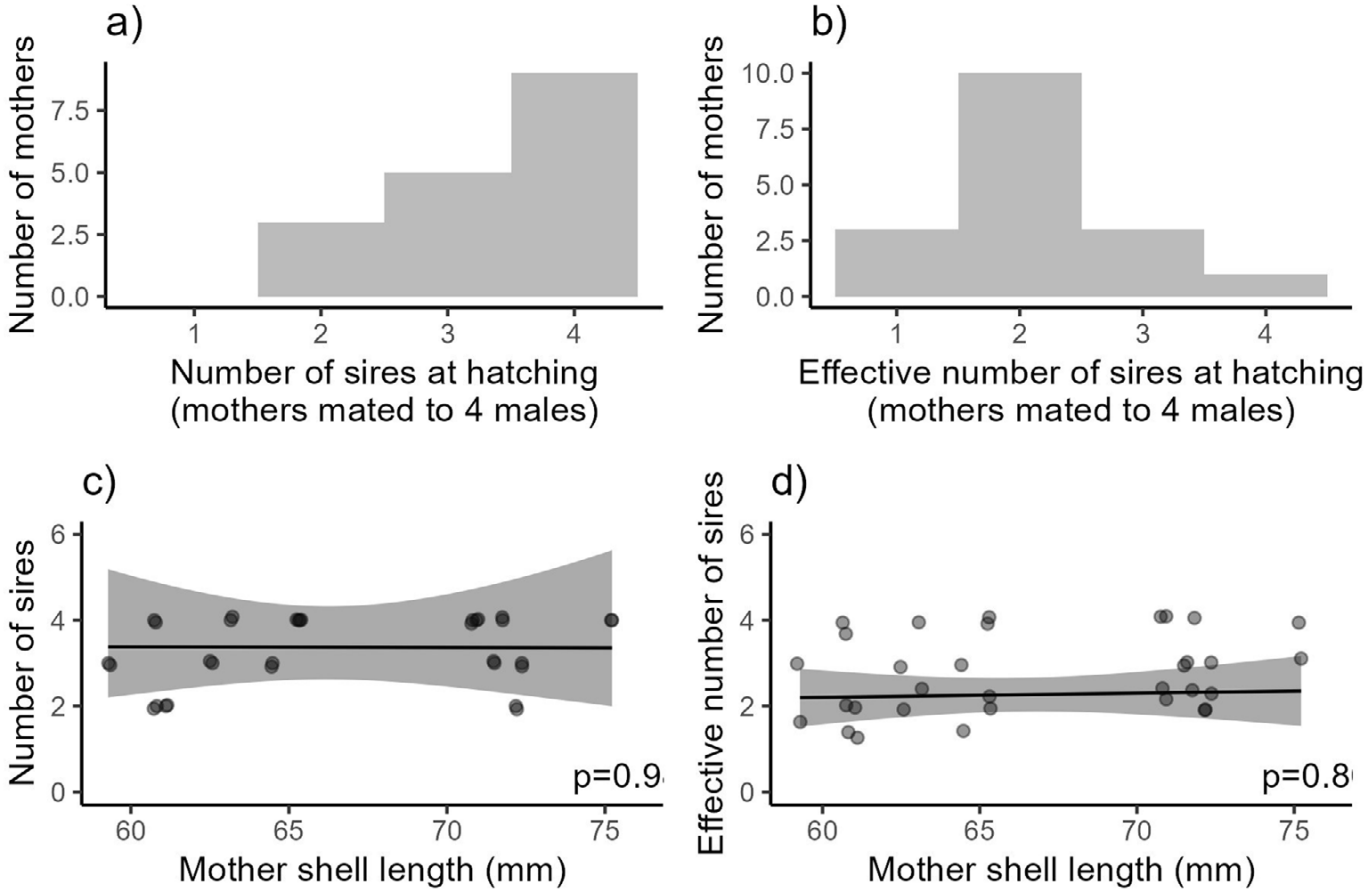
Histogram showing (a) the distribution of the number of sires per mother and (b) the effective number of sires per mother. The effect of mother length (mm) on (c) number of sires and (d) effective number of sires.

**FIGURE 6:**
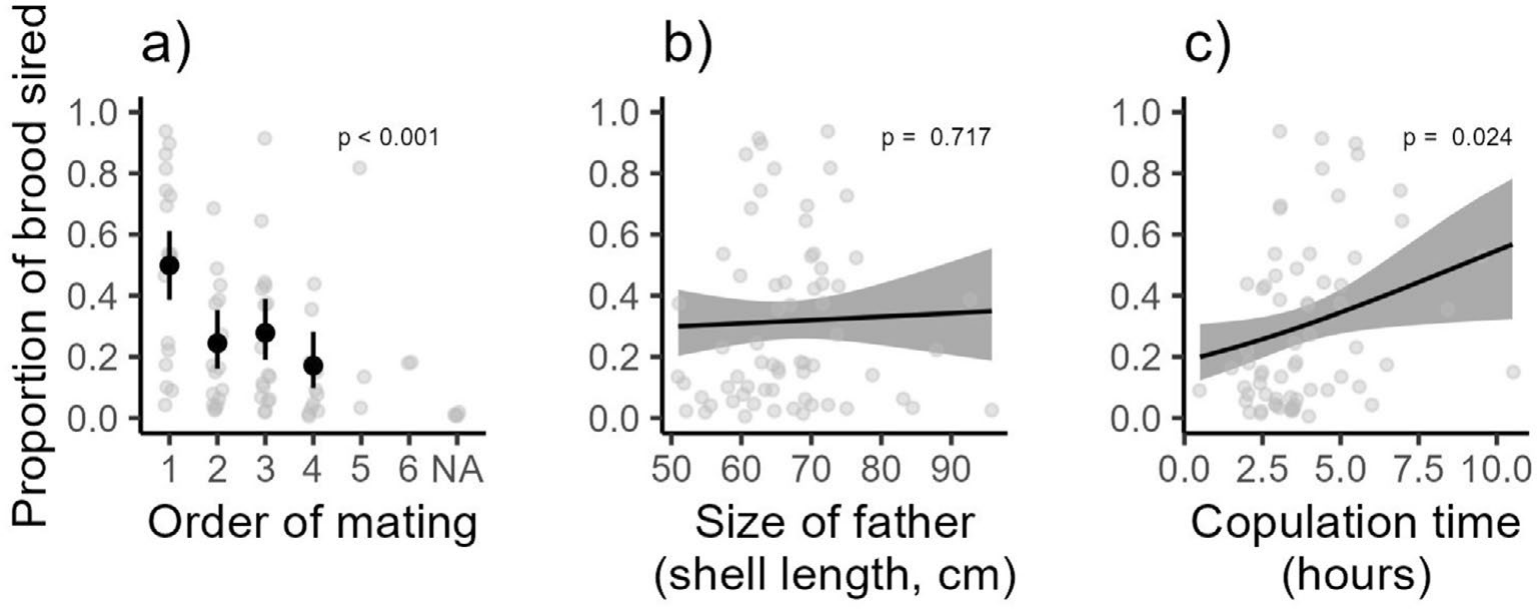
The proportion of offspring sired by males based on (a) the order in which they were mated, (b) the size of sire (cm), and (c) the time in which male and female pairs copulated.

For females mated to four males, the mean number of sires at hatching was 3.35 (*t*_16_ = −3.39, df = 16, *p* = 0.002; Figure 5a). The effective number of sires per brood averaged 2.3, significantly fewer than the number of sires detected (*t*_37_ = 3.58, *p* < 0.001), indicating an uneven paternity share among successful males (Figure 5b). The effective number of sires did not affect female reproductive output (*χ* ^2^ = 0.056, df = 1, *p* = 0.813), while female length significantly influenced the number of offspring produced (*χ* ^2^ = 6.37, df = 1, *p* = 0.012). Female size did not impact the number of sires present (*χ* ^2^ = 0, df = 1, *p* = 0.985) or the effective number of sires within a brood (*χ* ^2^ = 0.062, df = 1, *p* = 0.804; Figure 5d). In mixed brood treatments, the number of offspring a male sired was significantly affected by mate order (*χ* ^2^ = 16.94, df = 3, *p* < 0.001; Figure 6a) and copulation time (*χ* ^2^ = 5.09, df = 1, *p* = 0.024; Figure 6c), but not male size (*χ* ^2^ = 0.13, df = 1, *p* = 0.717; Figure 6b). First‐mated males and those with longer copulation times sired a greater proportion of offspring than other males.

## 4 Discussion

Multiple mating is a widespread phenomenon that can influence the genetic and phenotypic composition in the next generation, population viability, and adaptation to heterogeneous environments (Zeh and Zeh 2001; Tarpy 2003; Lotterhos 2011; Holman and Kokko 2013). While there are many hypotheses disentangling direct and indirect benefits of polyandry, all generally predict that polyandry ultimately leads to the production of more offspring per brood, either through fecundity or offspring viability (Arnqvist and Nilsson 2000; Jennions and Petrie 2000; Byrne and Whiting 2008; Noble et al. 2013). However, we found that there were no changes in the reproductive output or survival to hatching based on the number of males we experimentally presented to females for copulation. Furthermore, genetic markers indicated fewer sires at hatching than the number of mates and provided evidence for paternity skew at hatching, where paternity share declined with mate order and increased with copulation duration. These results suggest that common explanations for polyandry that have received considerable support in other taxa do not have support in this system, necessitating other explanations.

There are several possible explanations for why the benefits of polyandry were not detected. One hypothesis is that any genetic benefits manifest after offspring hatching. For instance, the benefits of polyandry that manifest after offspring development have been detected in insects (Sakaluk et al. 2002), birds (Gerlach et al. 2012), and mammals (Klemme et al. 2008). However, early developmental stages are when mortality is highest in most marine invertebrates, and fitness is often strongly correlated with early‐life traits such as larval size (Marshall et al. 2003), suggesting that the benefits of polyandry for offspring viability are likely to be realized at hatching size (McLeod and Marshall 2009).

While the degree to which survival and reproduction after hatching shapes polyandry in crown conch remains to be tested, we still expected to detect higher phenotypic variance and offspring survival in early life stages in broods from multiple matings compared to single matings, if these effects were present at all. Mean offspring size at hatching did not differ. However, variation in offspring size at hatching within egg capsules from mothers mated to multiple sires was greater than mothers mated to a single male. Previous empirical studies have also found greater size variation in mixed brood scenarios (Brown et al. 1996; Saidapur and Girish 2001; Gerlach et al. 2007). Greater size variation at hatching for polyandrous mothers could indicate that greater genetic diversity in a brood leads to greater phenotypic variation within broods, such as greater variation in egg size or development rate. In these cases, increased phenotypic variation could lead to decreased competition among unrelated siblings through differential resource exploitation (e.g., oxygen consumption) (Kamel and Williams 2017). Greater size variation within a brood could also be caused by sire identity, where certain fathers produce larger offspring compared to other fathers in the same brood and are more likely to be sampled under polyandry compared to monandry (García‐González and Simmons 2005; Crean et al. 2013). Regardless, these explanations might only explain size variation, and not mean size or number.

We also expected that female mate choice or sperm competition would lead to paternity skew if a genetically superior male sired the most offspring (Kupfernagel et al. 2010; McCullough et al. 2017). While we did detect paternity skewness, we cannot account for any effects of difference in sperm supply among males beyond assuming larger males provide more sperm and observing that male size did not affect paternity share. Nonetheless, we did find that the first male tended to dominate the brood of most females (Figure 6). Order precedence is consistent with the idea that male behaviors may influence paternity share, rather than outcomes driven by female cryptic choice or male genetic “quality”, though other hypotheses (e.g., bet‐hedging, encounter rates) do not necessarily predict such patterns. Longer copulation times were associated with a higher proportion of offspring sired, which may reflect increased sperm abundance or improved sperm placement during extended mating. Overall, these results are in contrast with other invertebrate studies that found that offspring diversity led to greater offspring survival (Byrne and Roberts 1999; Ivy and Sakaluk 2005; Crean et al. 2013; Ameline et al. 2023).

Another hypothesis for why the benefits of polyandry for females were not detected is that, instead of increasing arithmetic mean fitness within a generation, polyandry may have evolved as a bet‐hedging strategy that decreases variance in fitness across generations (Hughes and Boomsma 2004; Yasui and Garcia‐Gonzalez 2016; Yasui and Yamamoto 2021). In that case, benefits may not be detected within a single generation, and experiments across multiple generations would be needed (García‐González et al. 2015). However, bet‐hedging via multiple mating seems unlikely in most populations, including our focal population, because females often mate with a different set of males (Hooks and Burgess 2024), thereby reducing fitness correlations among individuals and the scope for bet‐hedging to evolve (Starrfelt and Kokko 2012; Holman 2016). When different females mate with different males, any risks of reduced offspring viability are already spread across monandrous females, making a polyandry strategy only likely to evolve in small populations (Starrfelt and Kokko 2012; Holman 2016). Bet‐hedging is more likely when the same set of males contribute offspring to multiple polyandrous females. Our previous work estimating polyandry in the field found very little evidence that the same male sired offspring of multiple females (Hooks and Burgess 2024).

Furthermore, we detected unequal paternity share in polyandrous broods, limiting the potential for multiple mating to proportionally increase genetic diversity of offspring. Without multiple sires being represented at similar levels in a brood, any benefits that come with greater genetic diversity are reduced, leading to the inference that benefits from increased genetic diversity are not always maximized in polyandrous mating systems (Lotterhos 2011).

Our results leave us with the question of why females mate multiply, if not for the reasons hypothesized. Multiple mating may be a consequence of sexual selection where multiple mating benefits males more than females (e.g., Bateman 1948; Brown et al. 2004; Wright et al. 2013; Boulton et al. 2018) or the act of refusing a male is more costly than mating itself (i.e., convenience polyandry; Rowe 1992). For the latter, polyandry benefits females by reducing the costs of sexual harassment. Cases of convenience polyandry have been documented in taxa where males cause harm to the female when mating is refused (Crudgington and Siva‐Jothy 2000), when the act of refusing males increases the risk of predation (Han and Jablonski 2010), or the time it takes a female to refuse a male takes too much time away from foraging (Rowe 1992). Similarly, multiple mating may just reflect encounter rates with males when finding mates is difficult or there is a patchy distribution of mating partners (Kokko and Mappes 2012). In female crown conch, we hypothesize that mating is costly based on behavioral observation of mating in the lab and in the field (Hooks, personal observation). We found that mating pairs can copulate for hours (10 h or more, with the average at 3–4 h), females do not feed during mating, and mating pairs remain immobile which increases predation risk (Hooks, personal observation). In the field, several males are often observed crowding around and piling on females, each trying to mate. Similarly, larger males attempting to mate with smaller females can increase their access to mating (Hooks, personal observation). In each of these cases, female refusal might come at an energetic cost that may not outweigh the cost associated with mating. Also, males will copulate with females while females are laying egg capsules; therefore, females refusing mating during egg laying could result in a reproduction cost if the female must stop laying in order to refuse a mate (Hooks, personal observation). Nevertheless, even if females are mating multiply due to convenience or by chance, multiple sires were not represented equally in the brood. Instead, successful males that sire the most offspring are the ones that mate first or copulate the longest, presumably contributing the most sperm. Future work should formally test whether convenience polyandry is operating in this system (Boulton et al. 2018).

In summary, our experimental approach found that females with a higher number of mates: (1) did not produce more offspring, (2) did not produce larger offspring at hatching, (3) did produce broods with greater variation in size at hatching, and (4) produced broods with a greater number of sires, but paternity skew meant that the number of sires was less than the number of mates. To make progress identifying why females mate multiply when direct or indirect benefits were not detected, future research should include quantifications of mating cost versus the cost of refusal of mating.

## Author Contributions

**Alexandra P. Hooks:** conceptualization (equal), data curation (lead), formal analysis (equal), funding acquisition (lead), investigation (lead), methodology (equal), project administration (lead), visualization (equal), writing – original draft (lead), writing – review and editing (equal). **Scott C. Burgess:** conceptualization (equal), formal analysis (equal), funding acquisition (supporting), methodology (equal), resources (equal), supervision (supporting), validation (equal), visualization (equal), writing – original draft (supporting), writing – review and editing (equal).

## Ethics Statement

**S**pecimens were sampled under a Florida Fish and Wildlife Conservation Commission Special Activity License SAL‐16‐1651.

## Conflicts of Interest

The authors declare no conflicts of interest.

## Data Availability

Data and R code to reproduce the analyses and figures can be found in the Dryad Digital Repository: DOI: 10.5061/dryad.7h44j104q.
